# EpiViewer: an epidemiological application for exploring time series data

**DOI:** 10.1186/s12859-018-2439-0

**Published:** 2018-11-22

**Authors:** Swapna Thorve, Mandy L. Wilson, Bryan L. Lewis, Samarth Swarup, Anil Kumar S. Vullikanti, Madhav V. Marathe

**Affiliations:** 1 Department of Computer Science, Virginia Tech, Blacksburg, Virginia, USA; 2 Network Dynamics and Simulation Science Laboratory, Biocomplexity Institute of Virginia Tech, Blacksburg, Virginia, USA; 3 Department of Computer Science, University of Virginia, Charlottesville, Virginia, USA; 4 Biocomplexity Institute, University of Virginia, Charlottesville, Virginia, USA

**Keywords:** Epidemiology, Visualization, Temporal, Time series, Metrics, Line chart, Bar chart, User actions

## Abstract

**Background:**

Visualization plays an important role in epidemic time series analysis and forecasting. Viewing time series data plotted on a graph can help researchers identify anomalies and unexpected trends that could be overlooked if the data were reviewed in tabular form; these details can influence a researcher’s recommended course of action or choice of simulation models. However, there are challenges in reviewing data sets from multiple data sources – data can be aggregated in different ways (e.g., incidence vs. cumulative), measure different criteria (e.g., infection counts, hospitalizations, and deaths), or represent different geographical scales (e.g., nation, HHS Regions, or states), which can make a direct comparison between time series difficult. In the face of an emerging epidemic, the ability to visualize time series from various sources and organizations and to reconcile these datasets based on different criteria could be key in developing accurate forecasts and identifying effective interventions. Many tools have been developed for visualizing temporal data; however, none yet supports all the functionality needed for easy collaborative visualization and analysis of epidemic data.

**Results:**

In this paper, we present EpiViewer, a time series exploration dashboard where users can upload epidemiological time series data from a variety of sources and compare, organize, and track how data evolves as an epidemic progresses. EpiViewer provides an easy-to-use web interface for visualizing temporal datasets either as line charts or bar charts. The application provides enhanced features for visual analysis, such as hierarchical categorization, zooming, and filtering, to enable detailed inspection and comparison of multiple time series on a single canvas. Finally, EpiViewer provides several built-in statistical Epi-features to help users interpret the epidemiological curves.

**Conclusion:**

EpiViewer is a single page web application that provides a framework for exploring, comparing, and organizing temporal datasets. It offers a variety of features for convenient filtering and analysis of epicurves based on meta-attribute tagging. EpiViewer also provides a platform for sharing data between groups for better comparison and analysis. Our user study demonstrated that EpiViewer is easy to use and fills a particular niche in the toolspace for visualization and exploration of epidemiological data.

**Electronic supplementary material:**

The online version of this article (10.1186/s12859-018-2439-0) contains supplementary material, which is available to authorized users.

## Background

In the face of an emerging epidemic, like the Ebola outbreak in West Africa in 2014 or the Zika outbreak in Brazil in 2017, authorities often turn to epidemiologists to help determine the likely severity of the outbreak and to identify strategies to curtail the spread of the disease. Epidemiologists have a number of approaches they can use to assess the situation, including reviewing historical outbreaks and strategies that have been tried in the past; however, visualization of different kinds of spatiotemporal datasets are key in interpreting the scope of the outbreak [1].

However, sometimes the review of time series data is not straightforward. During the Ebola crisis, for example, epidemiologists from many organizations were tasked with identifying measures likely to be effective in stopping the spread [2, 3]; this required a good understanding of the spread and prevalence of the infection, as well as the likely progression if left unchecked. There were a number of sources for surveillance data, including local government tallies and statistics provided by the World Health Organization (WHO) [4–6]. Meanwhile, several public health agencies and university laboratories offered forecasts of how Ebola was likely to progress in those regions, including the Centers of Disease Control and Prevention (CDC), Columbia University, the Laboratory for the Modeling of Biological Socio-technical Systems (MoBS Lab), and the Network Dynamics and Simulation Science Laboratory (NDSSL); these forecasters also released frequent updates to these datasets as new surveillance data surfaced, in order to provide policymakers with the most current information [5, 7–9].

As the researchers attempted to evaluate these datasets, however, they found that discrepancies in the data, aggregation type, data formats, category, and scope made it difficult to tell a cohesive story from the various datasets. Some of these problems were rooted in how the data was collected, including incomplete or overestimated reporting of the surveillance data, as well as different modeling methods for the forecasts [5, 10]. More fundamentally, however, there were inconsistencies in how the time series were reported (i.e., as incidence or cumulative counts), differences in the criteria measured (cases, hospitalizations, or deaths), varied reporting dates and frequencies, as well as differences across regions [10]. The sheer number of datasets to evaluate was an additional complication, especially because the datasets were often published in incompatible formats (such as Excel vs. PDF); this made it difficult to compare trends across datasets or to identify outliers or unreliable time series. A number of tools (Excel, R, and SAS) are used by epidemiologists to address these issues, however, they do not solve the fundamental issue of standardizing formats and allowing open access to these data. Additionally, as the ad-hoc team responding to this crisis was international, a persistent, standardized, and open way of visualizing and sharing these data was needed.

**Fig. 1:**
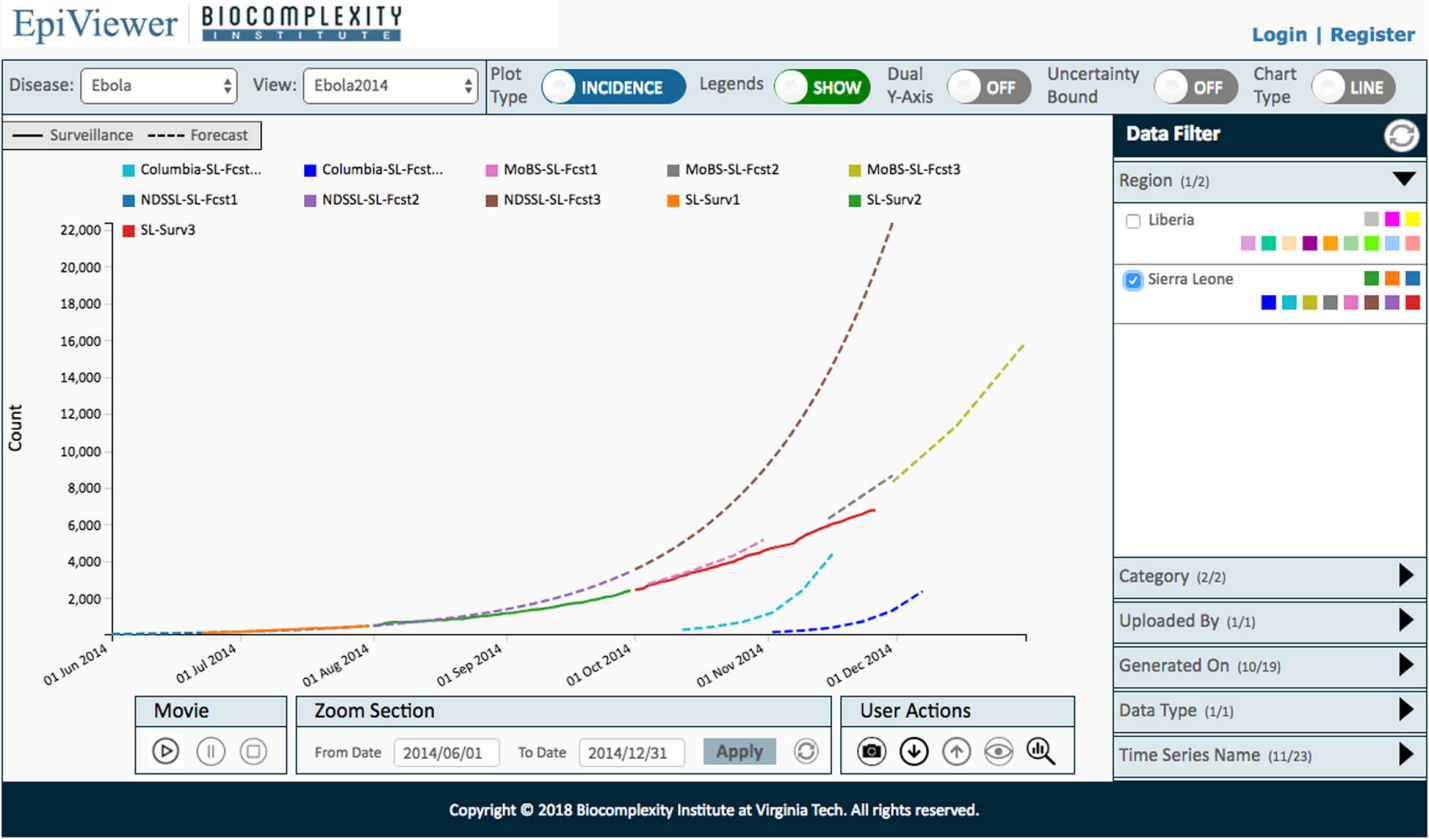
2014 Ebola outbreak graphs from Sierra Leone. EpiViewer was originally developed to help epidemiologists review time series data for the 2014 Ebola outbreak. The forecasts generated by the MoBS laboratory (pink, grey, and olive green) and some generated by NDSSL (blue and purple) were ultimately found to be close to the actual ground truth data (solid orange, green, and red)

We developed EpiViewer, a web-based time series visualization tool, to address these needs and enable researchers and policy-makers to evaluate these data (Refer to Fig. 1). Users can easily load time series data from disparate data sources, either as comma-separated-value (CSV) files or via a web services API, and view them as graphs on a common canvas; forecast data can also include Uncertainty Bounds (margins of error). EpiViewer offers a variety of different visualization options, including incidence vs. cumulative displays and the ability to use dual Y-axes to compare graphs of differing orders of magnitude. EpiViewer offers two graphing formats for viewing data: in a temporal fashion via line charts, or as bar charts to better evaluate the cumulative effect. Users assign metadata attributes to their time series, which EpiViewer, in turn, leverages to provide advanced filtering capabilities to limit which time series are visible on the canvas at a given time. Time series datasets can also be organized into workspaces, called *Views*, to allow users to group data in meaningful ways, such as separating epidemic data by year. Furthermore, users can make these views public in order to facilitate collaboration between researchers. Finally, time series data can easily be downloaded from EpiViewer, either as a csv file or via the web services API, so it can be loaded into other tools for data analysis.

In addition to facilitating visualization and distribution of time series data, EpiViewer also provides calculations of Epidemic features (Epi-features). Epi-features are statistical characteristics of an outbreak that can help researchers interpret the quality of the epidemic curves within a View, and to identify outlier time series [11]. The Epi-features provided by EpiViewer are described below:

*Peak time and value:* Peak value is the highest infection count over the course of the epidemic time series. The date when the peak value occurs is called the peak time.

*Total count:* Total count is the total (cumulative) number of infections over the duration of the time series. *First take-off time and value:* Some infectious diseases, like Dengue, start out almost dormant in the beginning, then suddenly exhibit a sharp increase in the number of cases just as the season commences.

Given EpiViewer’s powerful data filtering capabilities, it could also be a valuable addition to larger web-based systems as an integrated plug-in application. An example of this is the integration of EpiViewer into the Biosurveillance Ecosystem (BSVE), a large-scale analytics platform funded by the Defense Threat Reduction Agency (DTRA) for the analysis, visualization, and curation of real-time global epidemic and outbreak data. BSVE has a repository of data sources collated by DTRA, Los Alamos National Laboratories, and others [12, 13]. While BSVE offers applications customized to provide visualizations and analytical methods for specific data sources within its repository, EpiViewer allows users to compare data across multiple data sources along with their own data, which can lead to a more complete view on how an epidemic is progressing.

## Implementation

EpiViewer is developed using a three-tiered architecture, as explained in more detail below. Currently, there are two deployment options for this application:

***Standalone version:*** This deployment option offers EpiViewer as a standalone web application that can be run in a web-browser. It is an independent instance with its own database, and is not directly connected to any other application.

***Integration with the BSVE:*** EpiViewer is incorporated in the Analyst Workbench of the BSVE. This implementation offers the functionality of the standalone version as well as additional features that allow coordination with various BSVE components and data sources.

In addition to the architecture, there are two other implementation features of note: the calculation of the First take off Time and Value (Epi-feature) metrics, and assignment of time series to axes in the dual Y-axes view.

### Architecture

**Fig. 2:**
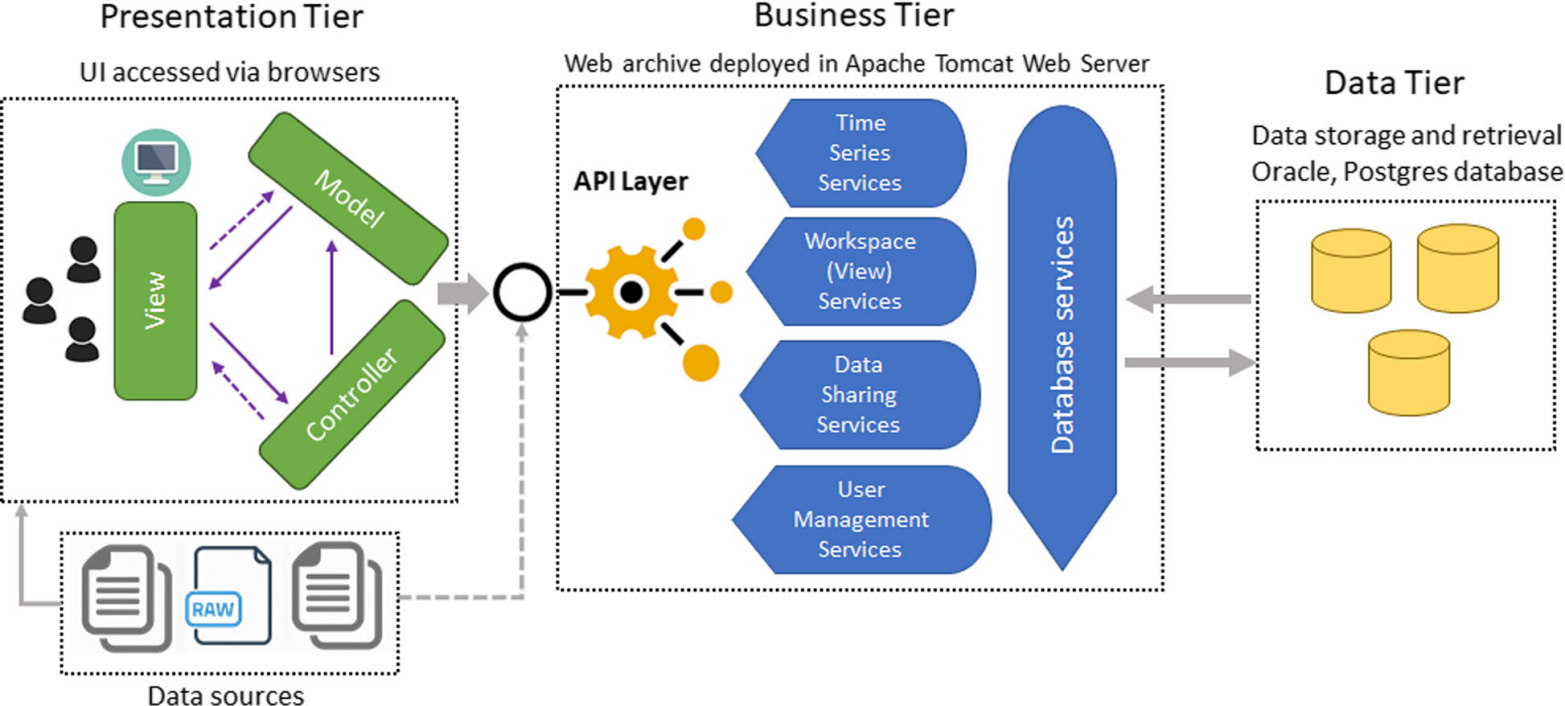
Architecture of EpiViewer. EpiViewer is developed as a three-tiered architecture. The Presentation Tier includes the user interface and application functionalities, and communicates with the business tier via an API layer. The Business Tier contains the core logic of all major application functionalities (e.g. upload data, data sharing). The Data Tier consists mainly of the relational database storage. Data can be loaded into the system via the user interface or externally via services from the API layer

The system architecture of the application is made up of three components: the presentation tier, business tier, and data tier, as shown in Fig. 2.

***Presentation Tier:*** EpiViewer is a Single Page Application (SPA) implemented using a model-view-controller architecture. An SPA is a web application that loads a single HTML page that is dynamically updated as the user interacts with the application. The presentation tier is implemented using HTML5, CSS3, Javascript, JQuery, AJAX, and D3. The controller uses AJAX to communicate with the API Layer, allowing parts of the page to be refreshed without the overhead of reloading the entire page.

***Business Tier:*** The business tier supports service-oriented computing by using Representational State Transfer (REST) APIs for data transfer. This request-response architectural style involves communication with a specific application service by sending all requests for that service to a specified endpoint. These endpoints consider data and functionality as resources and are accessed using Uniform Resource Identifiers (URIs), typically implemented as web links [14]. We use the Jersey RESTful Web Services framework [15], an open source framework for developing RESTful Web Services in Java. Entity management and the database service layer are managed using the Hibernate Java framework. Data formatted in JavaScript Object Notation (JSON) is used for communication between the tiers.

***Data Tier:*** EpiViewer uses a relational database for data storage. The application currently supports both PostgreSQL and Oracle.

EpiViewer can be viewed as a web-service application using a combination of resource-oriented and service-oriented architectural styles. This architecture style facilitates reusability and ease of interconnection with other systems. This style supports the interoperability of services by abstracting service details from the end-user application. This facilitates and increases vendor diversity options. An example of this is the integration of EpiViewer into the BSVE analytical framework.

### Calculation of first take off time and value

**Fig. 3:**
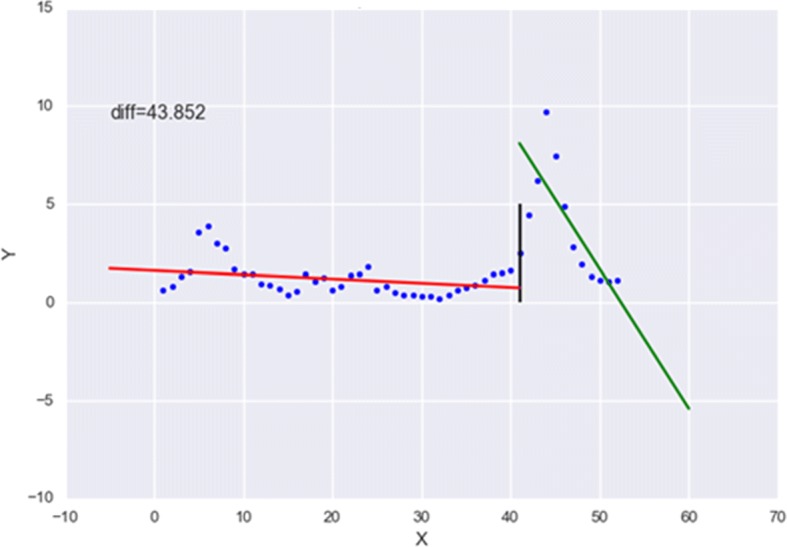
Depiction of the piecewise regression method utilized for calculating the Epi-feature ‘First Take-off Point’. In this illustration, the blue dots represent time series points on the epicurve; the black line indicates where the partition is for the current iteration; and the red and green lines indicate the line segments fitted to the two intervals

According to Tabataba et al. [11], “Mathematically, first take-off is the time at which the first derivative of the epidemic curve exceeds a specific threshold”. The first take-off threshold value typically depends on the type of disease and the outbreak severity, so this threshold is normally established by domain experts. However, as a web application that allows users to add new diseases, it is not reasonable to expect domain experts to establish the threshold values for every disease added to the system; instead, we use a piecewise linear regression approach to determine the first take-off value and time. Piecewise linear regression, or “broken-stick regression”, is a method of regression analysis in which the independent variable is partitioned into intervals, and a separate line segment is fit to each interval. When applied to epidemic curves [16, 17], this technique can be useful for identifying the time when an epidemic first takes-off. Figure 3 illustrates the process of partitioning the data points and applying the linear fit to the partitions.

The procedure for calculating the first take-off time is described below:

1. Let *T* be a time series having *n* records, where *T*_*i*_ is the *i*^*th*^ <*date*,*value*> tuple.
2. Sort all the *n* records of *T* in ascending order by date.
3. Partition the data into 2 segments such that the left partition is $\left [T_{1},...,T_{split\_index}\right ]$ and right partition is $\left [T_{split\_index+1},...,T_{n}\right ]$. Then, for each partition, find the best linear fit and record the sum of squared errors (SSE). For every partition, record
  <*date*_*partition*_,*value*_*partition*_,(*SSE*_*left*_−*SSE*_*right*_)>. Repeat this step until all possible partitions of the data have been processed such that the split_index goes from 2 to *n*−1.
4. Choose the minimum SSE value from the list. The data and value associated with this SSE value is the first take-off time and value.

### Assignment of time series to dual y-axes

When graphing multiple time series on a single canvas, differences in orders of magnitude between the time series (i.e., between cases and deaths) can effectively cause one time series to be “flattened”, which can complicate identification of trends. To address this issue, EpiViewer provides the option of splitting time series across dual y-axes; assignment is performed using the following steps:

1. Fetch the time series data from the database. This acts as the source data.
2. Calculate the maximum value across all the time series on the canvas from the source data. (This maxima is recalculated every time a time series is added to the view.)
3. Divide the overall maxima (derived in Step 2) by 2.
4. The maxima is now calculated for each time series and compared with the value obtained in Step 3. If the time series maxima is less than the Step 3 value, then it is assigned to the left axis; otherwise, the time series is assigned to the right axis.

## Results

### Application features

**Fig. 4:**
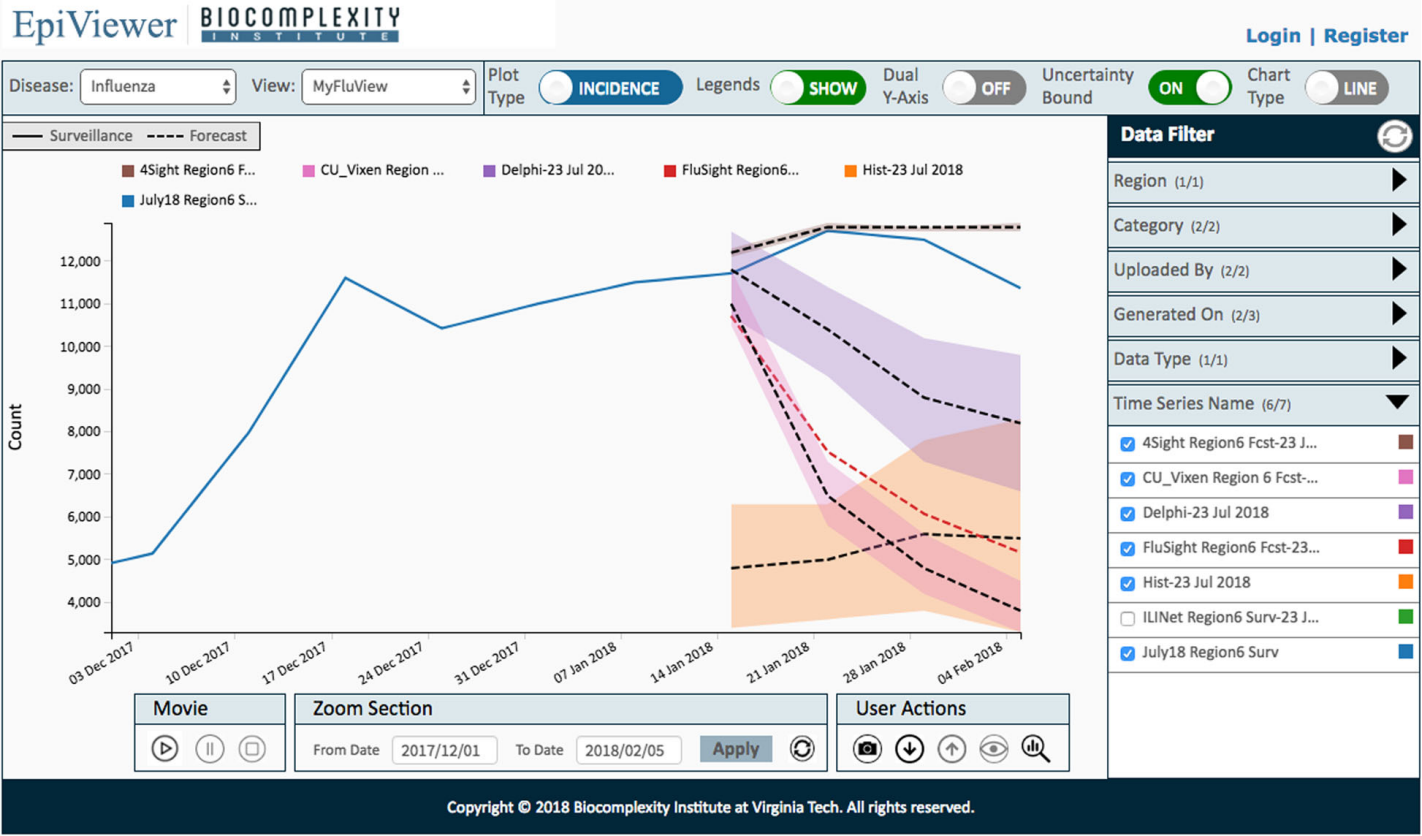
EpiViewer User Interface. The EpiViewer interface has 3 panels. The canvas panel is where the time series graphs from a workspace (view) are displayed. The user actions panel, located at the bottom of the canvas, allows users to perform operations on time series present in the canvas area, including upload time series, download View data, take a snapshot of the canvas, play a movie, zoom to a date range, view Epi-features for time series in the canvas, and manage Views (workspaces). The data configuration and filter columns are on the top and right-hand side of the canvas, respectively; they allow users to change the display options and control which time series are displayed on the canvas

The application features can be grouped across 3 panels: the canvas, data configuration and filters, and user actions. Refer to Fig. 4 to view a snapshot of the application.

The canvas panel is the area where the time series graphs for a given workspace (view) are displayed. Views are workspaces which allow users to organize their time series in logical ways. For example, a user who studies Influenza could create a view for each Influenza season instead of trying to crowd multiple seasons on the same canvas. Views are private by default, which means that the data is visible only to the owner, but users can also make their views public to allow other researchers to build on their collated data. Users can hover the mouse over the time series legends on the canvas to view Metadata and Epi-feature information for each time series. Users may also associate forecast time series graphs with surveillance time series so they appear on the canvas in the same color to imply a relationship; for example, the forecast may have been developed using the associated surveillance curve as input.

Through the data configuration and filter panels, EpiViewer offers a wide variety of display options and filtering capabilities to help researchers identify trends and make comparisons between time series that would be difficult to achieve through examination of standard chart data. The data configuration panel at the top of the page controls what dataset is displayed on the canvas through disease and view selection dropdowns. Further configuration of the canvas is achieved through the selection of plot type (incidence or cumulative), chart type (Line or Bar graphs), whether to display the time series across dual y-axes, and whether to display the legend. The uncertainty bounds option is used to view the margin of error data for the time series. The Filtering panel on the right allows users to change which time series curves are displayed on the canvas based on metadata attributes that were defined when the time series was uploaded, such as the region, whether the graph is Surveillance or Forecast, and the data type (i.e. Cases, Deaths, Hospitalization).

The user actions panel located at the bottom of the canvas allows users to interact with the time series present in the canvas area. From here, users can upload time series, download a zip file of all the time series in a View, take a snapshot (image) of the canvas to include in presentations, zoom to a date range within the View, review the Epi-features for time series curves on the canvas, edit Views, and play a movie. The movie feature allows users to watch as the time series are plotted on the canvas in order of the ‘Generated On’ date to assess how surveillance and forecast predictions have evolved as the epidemic has progressed; this can be especially useful when studying a volatile epidemic or for evaluating how epidemic predictions were made.

The solid blue line represents a surveillance curve for HHS Region 6. The other five forecast time series represent different teams that participated in the challenge. The error limits on four of these time series are visible since the ‘uncertainty bound’ option is selected. The curves have been filtered using the panel on the right hand side. A quick observation shows that the forecast generated by ‘4Sight’ team for HHS Region 6 is the most accurate forecast as compared to the others.

An example of data collected during 2014 Ebola outbreak from different sources such as World Health Organization, Columbia Lab, MoBS lab and NDSSL lab are displayed in Fig. 1. The forecasts produced by MoBS and NDSSL are better than the others for Sierra Leone. Other examples of the interface usability can be found in the Additional file 1.

### User study

We conducted a user study to assess EpiViewer’s ease of use. The participants (faculty, staff, and students at Virginia Tech) had not used EpiViewer before, and came from a variety of academic backgrounds, including computer science, epidemiology, and public health.

At the beginning of the user study, an instructor provided a brief overview of the application, including an explanation of the problem it was designed to solve. Users were then given an opportunity to try out the system by performing a checklist of 11 tasks covering the important utility functions of the application, like importing and filtering time series, and user actions like taking snapshots of the data; a complete list of the tasks are included in the Additional file 2.

**Fig. 5:**
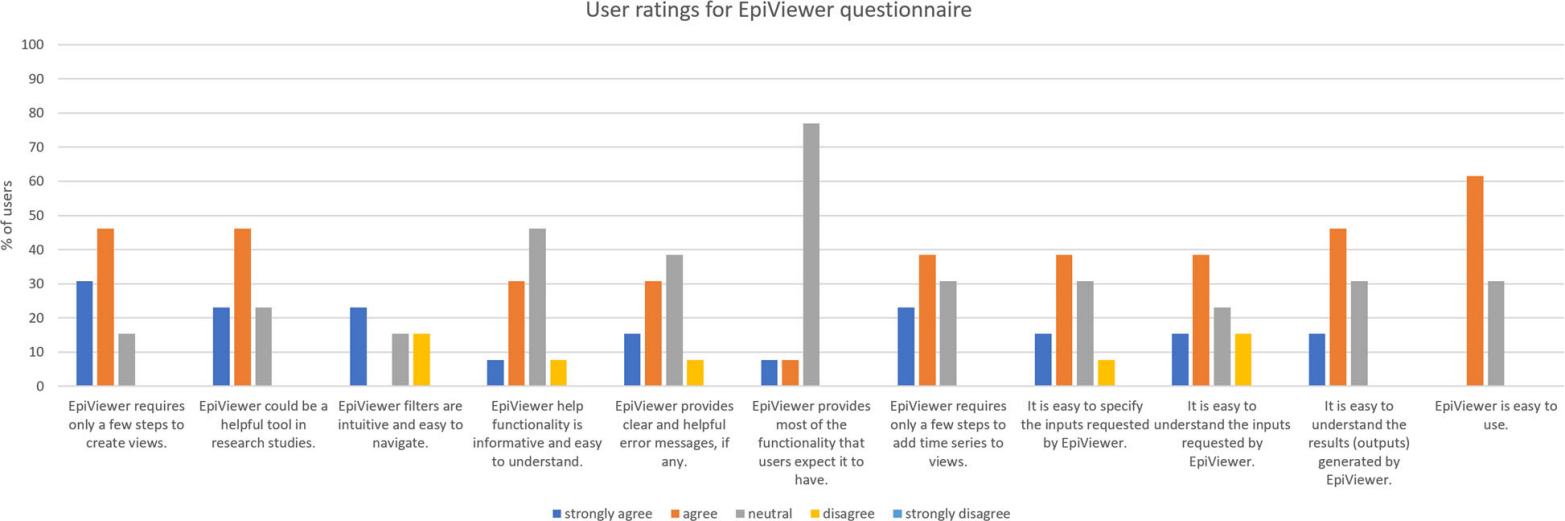
User ratings of various tasks performed on the user study

Both quantitative and qualitative data were collected over the course of the study. Quantitative data included the start and end times recorded for each task so we could assess how intuitive the application is. Qualitative data included handwritten observation notes from the instructors documenting the sequence of actions users took to complete major tasks like importing and filtering data, along with problems they encountered in performing the tasks. Participants recorded overall user experience via an online survey, which included prompts for ease of use, problems faced, and open-ended questions like applications for this tool and recommendations for improvements. Refer to Additional file 3 for further details. Refer to Fig. 5 for a breakdown of the participants’ reactions.

**Fig. 6:**
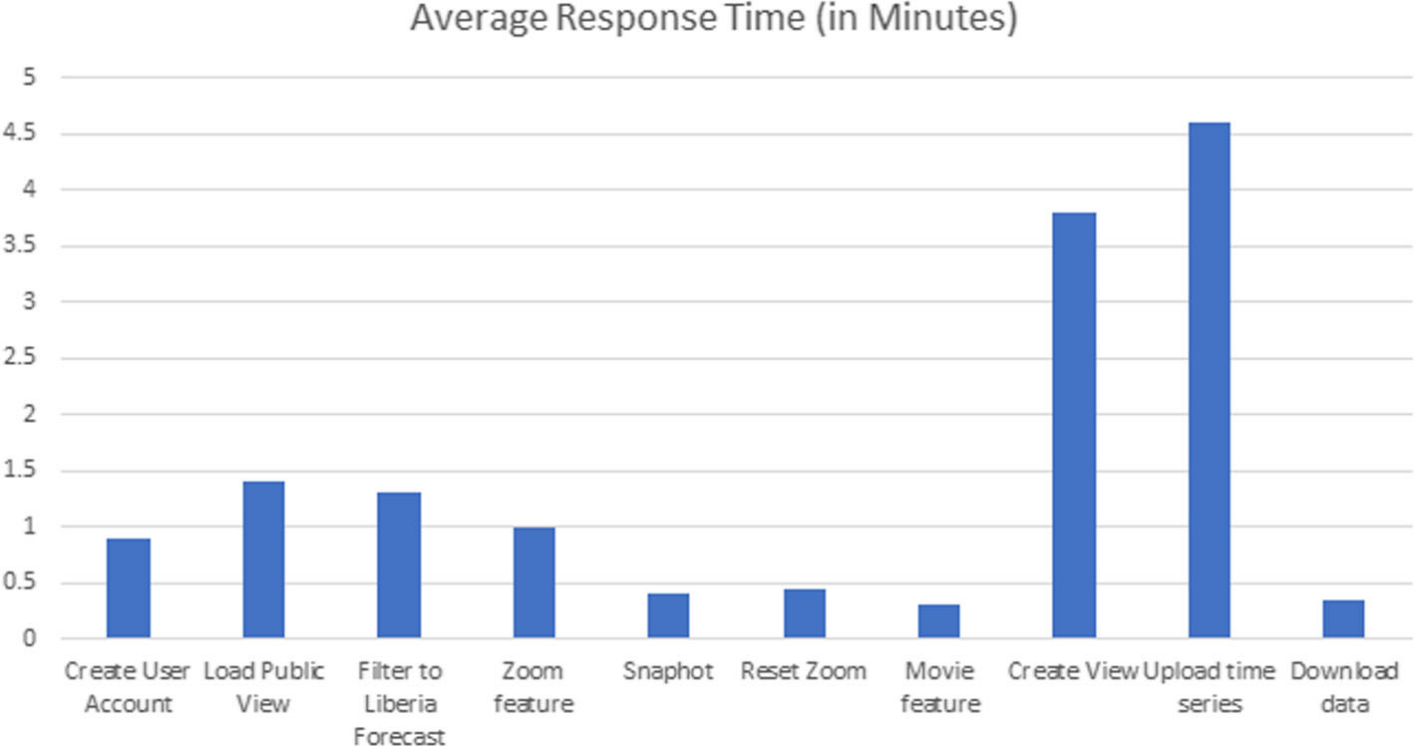
Average response times for performing assigned tasks in EpiViewer In the user study, participants were able to complete an assigned list of tasks in a reasonable span of time. The average total time spent on the 11 tasks was 24 min

We observed that 80% of the users were able to use the application without difficulty (Refer to Fig. 6). Important application functionalities like uploading data, user account creation, view creation and filtering were performed easily by users, and they were able to complete the tasks within the allotted time. They cited the ability for grouping datasets as views/workspaces and for visualizing data from different sources and attributes on one screen to be helpful features. Many felt that analysis and navigation of the time series were simplified by the metadata filters and zoom functionality. The application was found to have a quick learning curve overall. Users also communicated interest in using the application in other research areas for analyzing data feeds.

The participants did indicate that they felt that the distinction between public and private views and time series was unclear. They also requested more detailed feedback messages after performing important tasks like uploading datasets, zooming, and resetting filters. These suggestions were used to guide enhancement decisions for improving EpiViewer’s user interface.

## Discussion

### Benefits

One of the major benefits of EpiViewer is as a platform for sharing and comparing epidemic curves. Multiple articles [8, 10, 18] have highlighted the need for researchers to share data during a pandemic. Even when individuals and organizations are willing to share their data, the harder question they face is: how does one go about doing it? Simply putting it on a website does not facilitate comparison with data from other research organizations because there is no standard format for sharing that data. Usually, individual institutions publish their data in a format convenient to their specific systems. This makes re-usability that much harder, and ultimately leads to situations where the data is not shared effectively.

EpiViewer is a step towards addressing these challenges for temporal epidemiological datasets. With its easy-to-use interface for loading, publishing, and comparing data, EpiViewer is designed so that data from multiple parties can be shared and visualized in a straightforward manner, and executive reports can be constructed in an expedited fashion.

The implementation of Epi-features in EpiViewer allows users to evaluate the time series data from a statistical standpoint. Expert domain users can draw informed conclusions about the time series, especially in determining the quality of forecasts across different sources. The ‘First Take-off Time and Value’ Epi-features would normally require expert intuition to determine a threshold value for a given disease. This manual interference is eliminated by adopting the segmented regression approach.

EpiViewer was originally designed to be a lightweight, standalone web application, but has been enhanced to support easy integration within larger ecosystems; this is made possible because of the service-oriented computing style provided by the REST APIs to support interoperability of services. Refer to Additional file 4 for further details.

### Comparison with similar existing software systems

EpiViewer’s simple and intuitive web interface offers a time-effective way for scientists to upload and visualize their time series data. It natively offers a variety of filtering mechanisms and display features to enhance visual analysis of the data. Although these features are available in Excel, R, SAS, or Matlab, EpiViewer’s interface makes transitioning between different visualizations quick and seamless. These filtering mechanisms also enable comparisons between different data types of different scales (deaths, hospitalizations, cases, etc.) so that trends, correlations or anomalies can be swiftly perceived across the dataset. In addition, the application’s ‘Movie’ feature adds a temporal component to visualizing the data that would be harder to achieve in applications like Excel, R, SAS, or Matlab. The Additional file 2 contains a movie example.

The web-based platform also makes it easy for users to share their data with other scientists in a standard format. To achieve data sharing in R, Matlab or similar tools, the user has to write scripts to process the data, to visualize it, and then to share it via a CSV, PDF or image files. Even applications like Dotmapper [19] have reported the need for improved data sharing. Through EpiViewer’s public views, researchers can make their data available to other scientists for download, either in CSV format or via Rest APIs (JSON input and output), or even create a snapshot of the system simply by clicking a button.

EpiViewer is already configurable for integration within larger analytic systems (like the BSVE); this, along with the built-in REST APIs, can be used to automate the process of loading data from other data sources, either from within or outside of the parent system. Although this functionality could be achieved with R, it would require the development of custom scripts and interfaces compatible with the target system. With EpiViewer, the user need not worry about the implementation details of the services and API.

Another noteworthy web application is FluSight [20, 21]. Forecasting teams that participated in the CDC Flu challenge created FluSight in 2017 as a tool to visualize the CDC surveillance flu data and the forecasts submitted by the different teams. Although the teams collaborated outside of the application to share their models and forecasting data, the interface provides filtering by HHS region to help the viewer understand how the teams modeled the Influenza progression at different time stamps through current and past seasons. However, while FluSight offers visualization features that are essential for exploring epidemiological datasets, the website is built specifically for the U.S. CDC Flu Challenge, and the features are tailored to that challenge. Furthermore, users cannot upload and compare their own data within the system.

### Limitations

The system exhibits a few limitations. First, the canvas area looks cluttered if a view contains more than thirty graphs. Second, the application currently supports only two chart types - line chart and bar chart. It does not support other visualization motifs such as chloropleth maps, social network graphs, or phylogenetic trees. The application does not support multi-views combining different visualization motifs which could help to analyze data more effectively.

### Future work

Future plans for enhancing the application include:

***Spatial view:*** A heat map view that colors a geographical map based on the severity of the outbreak across the subregions would add a spatial aspect to epidemic analysis in addition to the existing temporal aspect. Users could then better identify trends on a geographic scale, and also identify hot spots where applying interventions could curtail the epidemic.

***Error measure metrics:*** In addition to the Epi-features, error measure metrics like Mean Absolute Error (MAE) and Mean Absolute Percentage Error (MAPE) [11] can be calculated for forecasts once ground truth data is available. These metrics will quantify the error across the duration of the forecasted time series into a single statistic, allowing the user to quantitatively understand the quality of the different forecasts.

***Advanced graph association:*** Currently, only one-to-one relationships can be established between surveillance and forecast time series through the Associated Graph feature. In real life applications, there may be multiple forecasts associated with a particular surveillance curve; being able to visualize how surveillance-related forecasts differ may make them easier to compare visually.

***EpiJSON support:*** EpiJSON is a proposed standard format for exchanging time series data between applications [22]. Integrating support for uploading and downloading data in EpiJSON format would be helpful for promoting adoption of EpiViewer in the epidemiological community.

## Conclusions

We present EpiViewer: a lightweight visualization framework for viewing and sharing, surveillance and forecast time series data. The framework facilitates exploring, comparing, filtering and organizing temporal datasets to allow researchers to conveniently manipulate time series through the use of meta-attribute tagging. Importantly, EpiViewer supports data sharing and computation of general epidemiological metrics for time series on the fly. Finally, EpiViewer can be configured to support easy integration within larger software systems. We believe that EpiViewer fills a particular niche in epidemic science.

## Availability and requirements

**Project name**: EpiViewer

**Project home page**: http://epics.vbi.vt.edu/epiviewer/index.jsp

**Operating system(s)**: Platform independent

**Programming language**: Java, Javascript, D3, and Oracle or PostgreSQL

**Other requirements**: Java 1.7.0 or higher, Tomcat 7.0 or higher

**License**: None required.

**Any restrictions to use by non-academics**: None.

## Additional files

Additional file 1 Application functionality. (PDF 883 kb)

Additional file 2 Exercise for EpiViewer Focus Group. (PDF 341 kb)

Additional file 3 List of Questions for EpiViewer Focus Group Evaluation. (PDF 193 kb)

Additional file 4 Application Web Services. (PDF 73 kb)

## Abbreviations

API: Application programming interface
BSVE: Biosurveillance ecosystem
CDC: Centers for disease control and prevention
CSV: Comma separated value, a flat-file format for exchanging data
DTRA: Defense threat reduction agency
HHS Regions: US department of health and human services regions are groupings of states used for aggregating epidemic activity
IRB: Institution review board
JSON: Javascript object notation
MoBS Lab: Laboratory for the modeling of biological socio-technical systems
NDSSL: Network dynamics simulation science laboratory
REST: Representational state transfer
SAS: Previously called “statistical analysis system”, sas is now the official name of this statistical software platform
SPA: Single page application
SPSS Statistics: Formerly known as “statistical package for the social sciences”
SQL: Structured query language
SSE: Sum of squares error
URI: Uniform resource identifier
