# Supplementary material for: EpiViewer: an epidemiological application for exploring time series data
Source: BMC Bioinformatics. 2018 Nov 22;19:449. doi: 10.1186/s12859-018-2439-0 (PMC6251172; doi:10.1186/s12859-018-2439-0)
Supplement: Supplementary file 1 — Application functionality. (PDF 883 kb) [file 12859_2018_2439_MOESM1_ESM.pdf]

Additional File : Application functionality and use cases

This document contains details of all the features of the applications and example use cases. The application is divided into 3 panels: Data Configuration and Filters, User Actions, and the Canvas. Figure 1 shows a snapshot of the application.

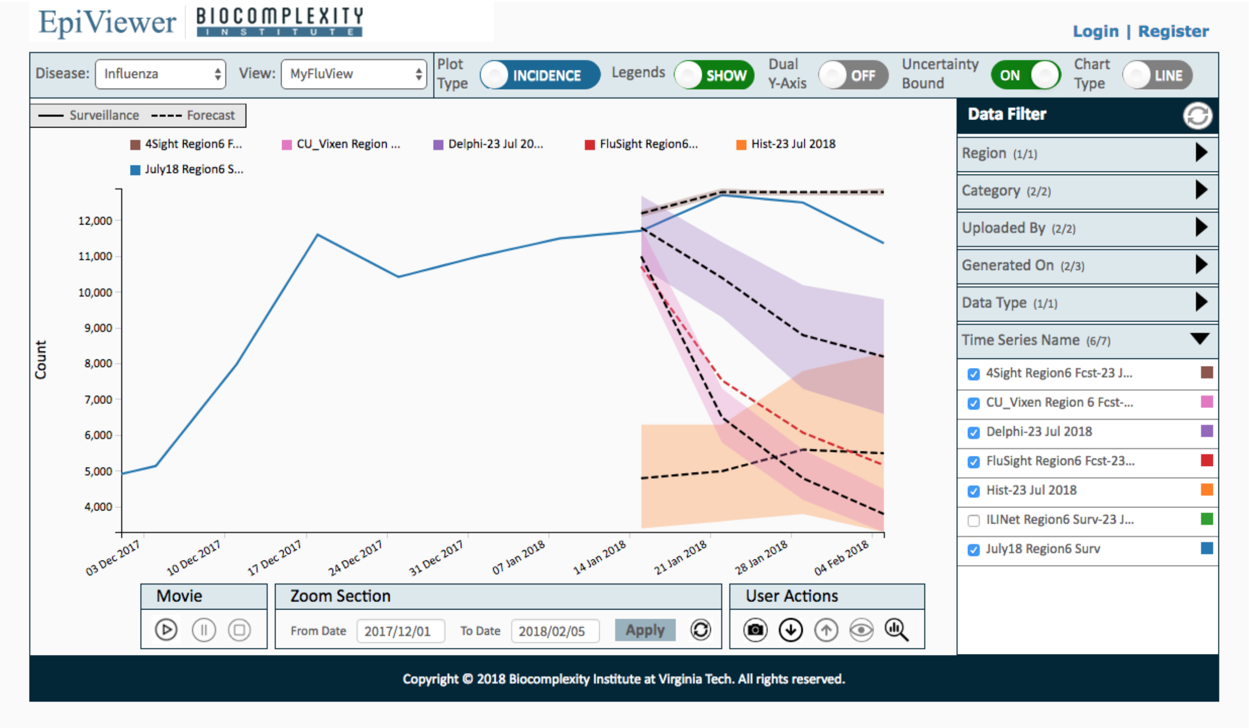

Figure 1: EpiViewer User Interface has 3 panels. The canvas is the area where time series graphs from a workspace (view) are displayed. The user actions panel located at the bottom of the canvas helps users perform multiple operations on time series present in the canvas area. These actions include upload time series, download view data, snapshot of the canvas, play a movie, zoom data, epi-features for time series in the canvas, workspace (view) functionalities. There are 2 data configuration and filter columns on the top and right-hand side of the canvas. The filters on the right facilitate drilling down using metadata attributes and the top data configuration panel configures the output in the canvas area.

Views: Views are workspaces which allow users to organize their time series in logical ways. For example, a user who studies Influenza could create a view for each Influenza season instead of trying to crowd multiple seasons on the same canvas. Views are private by default, which means that the data is visible only to the owner, but users can also make their views public to allow other

researchers to build on their collated data. Time series can be added to the views by importing comma-separated-value (CSV) time series files, or by copying time series from other views. Time series can also be removed from the views, either temporarily by filtering, or permanently via deletion.

Import data: Importing time series data from CSV files is one of the main features of the system. The file format for the data file should include a row for each data point in the series, with the date in the first column and data counts in the second column; if the data contains an uncertainty bound, the lower bound would be entered in the third column, and the upper bound would be included in the fourth column. A single row in an import file might look something like this:

10/17/2017, 237, 228, 245

When uploading a data file, users are prompted to define metadata attributes that apply to the time series, such as the time series name, whether the dataset is surveillance or forecast data (category), the region where the data was collected, whether the imported data is aggregated at the incidence or cumulative level (plot type), the timestamp when the data was obtained, etc.; this metadata can be used to filter the time series on the canvas. An additional metadata attribute is Associated Graph: a time series can be associated with another time series within that view to indicate a relationship between the datasets. Associated time series are displayed in the same color so researchers can easily identify those dependencies; the only constraint for graph association is that the two time series must be of different categories (i.e., surveillance time series can only be associated with forecasts, and vice versa.)

When EpiViewer is deployed within the BSVE, there are custom services for importing data from different BSVE data sources, including EpiArchive and the Event Trackers.

Canvas area: The canvas panel is where the time series graphs are displayed. Surveillance graphs appear on the canvas as solid lines, while forecasts are displayed as dashed lines. Metadata and epi-feature information for a particular epi curve can be viewed on the canvas by hovering the mouse over the legend entry; this action also highlights the curve in the canvas area for readability. When the mouse cursor is hovering over a data point on the selected curve, the curve is highlighted and a small pop-up shows information about the data point, including the data count and date for that entry.

In Figure 1, the canvas area shows six Influenza time series for HHS Region 6 of the United States. These time series are from the CDC Flu Challenge. The solid blue line represents a surveillance curve collected from the CDC website for the current season. The other five forecast time series are from different teams participating in the challenge. Four of these time series have uncertainty bounds. These bounds are visible since the 'uncertainty bound' option is selected. These curves are uploaded with uncertainty bounds in the csv format. These bounds give an idea of the variance/upper-lower error limits of the forecast. The curves have been filtered using the panel on the right hand side. A quick observation shows that the forecast generated by '4Sight' team for HHS Region 6 is much better than the other forecasts. While other teams' forecast data suggests that the Influenza spread was going to drop down, '4Sight' team forecast an upward trend. Also, one can see that the upper and lower error bounds for '4Sight' were smaller than the others, indicating lower error margins.

Data configuration and filtering capabilities

EpiViewer offers a wide variety of display options and filtering capabilities to help researchers identify trends and make comparisons between time series that would be difficult to achieve through examination of standard chart data.

Users start off by selecting the disease they wish to investigate from a drop-down list located at the upper left corner of the page; this selection will filter the dropdown list of views the user can choose from, which include those that are privately owned by the user as well as views that have been made public. The top panel section offers a ‘Plot type’ display option where users can choose to view the time series in either incidence or cumulative format. The user can also display the uncertainty bounds if margin of error data is available for their uploaded time series. Users can toggle between line and bar chart formats; these different perspectives can be useful for differentiating between the temporal aspect (line chart) as well as the cumulative (overall) impact of the disease (bar chart). Distribution of time series across dual y-axes based on order of magnitude is also available in this section via the ‘Dual Y-Axis’ option. Refer to Figures 2,3, and 4 for details. The dual y-axis option is handy for comparison for data having a wide range. For example, in Figure 4, it is useful to toggle the display to dual y-axis while comparing the time series trends between a smaller region (HHS Region 6) and a country (U.S.). We can now clearly see that the surveillance curves follow the same pattern/trend, but the forecasts for both the regions have different trends.

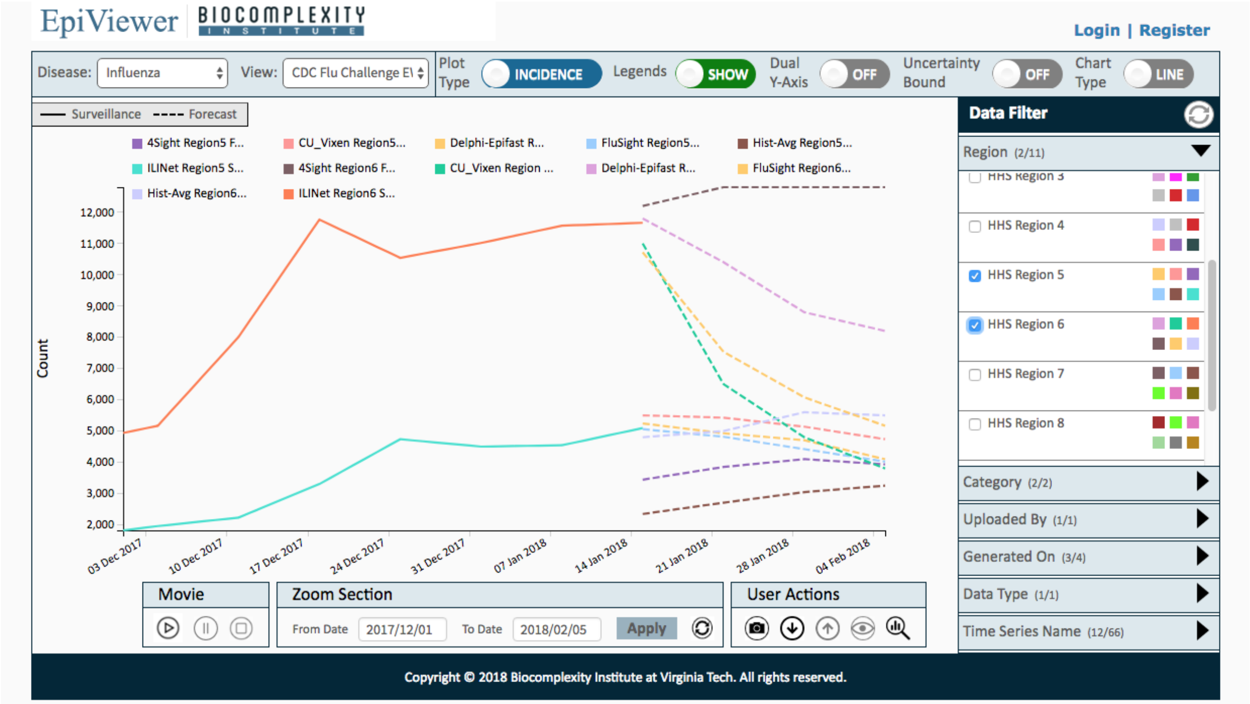

Figure 2: **Incidence line chart view.** When the Plot Type is set to “Incidence”, then the line chart displays the number of reported cases (or deaths, etc., depending on data type) on that date. This means that the line graph is likely to rise and fall with the epidemic activity.

Users can filter which time series appear on the canvas via data filters available on the right

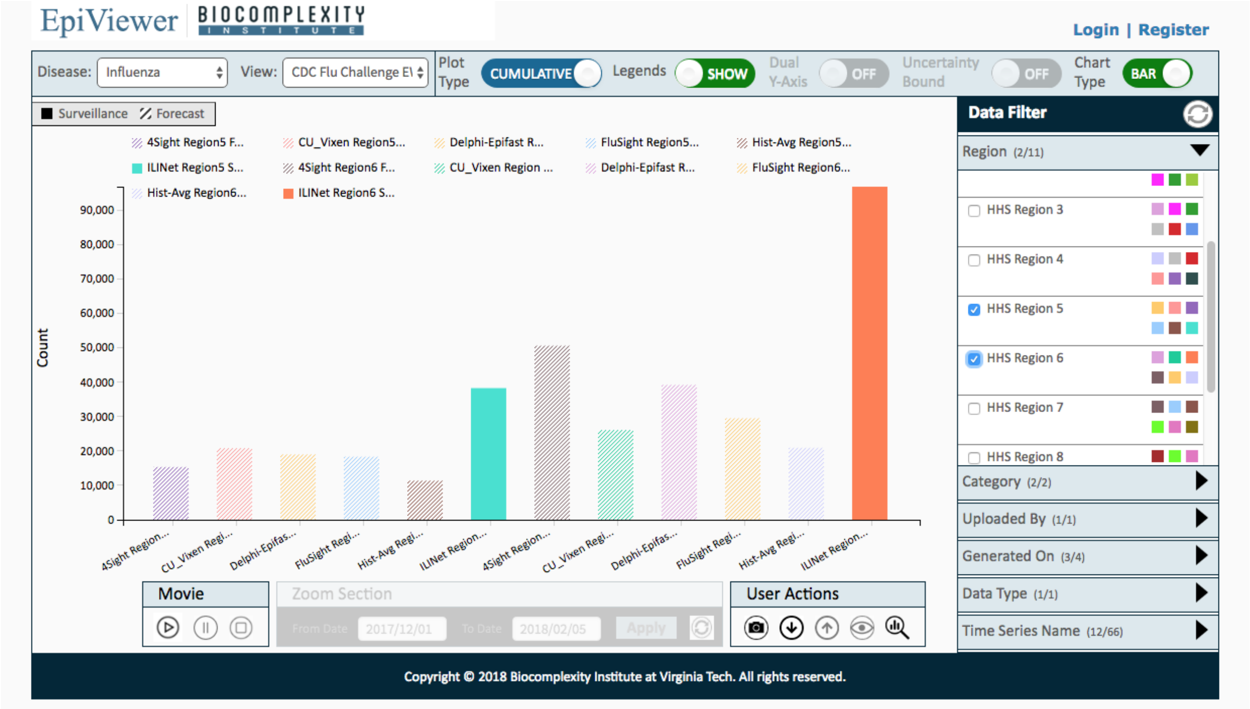

Figure 3: **Cumulative bar chart view.** When the Plot Type is set to "Cumulative", then each date would typically show the total number of cases (or deaths, etc.) as of that date. In line chart view, this would show the time series going up until possibly leveling out at the end of the epidemic; in bar chart view, the cumulative total is shown for each of the time series.

side of the EpiViewer display. The data filters rely on the metadata entered for each time series; they can be filtered based on Region, Category (Surveillance/Forecast), Uploaded By (owner of the original time series), Data Type (Cases, Deaths, or custom data types), Generated On (the date the surveillance data was collated or the forecast was predicted) and Time Series Name.

Users can reduce the scope of the canvas display by zooming in on a specific date range. This is done by clicking in the 'From Date' box of the 'Zoom Section' area at the bottom of the page; this will pop up a calendar widget where the user can select the desired start date. The user then repeats the process in the 'To Date' field to enter the end date for the zoom. Finally, the user can click on the 'Apply' button to zoom into the specified date range. When they want to return to the full canvas, they can click on the refresh icon to the right of the 'Apply' button.

User actions

This section describes the actions that users can perform on time series present in the canvas area.

Movie: The movie feature allows users to watch as the plots are laid out on the canvas in order of Generated Date so users can see how surveillance and forecast predictions have evolved over time. This can be especially useful when investigating a volatile epidemic, or if evaluating how epidemic predictions are made. To begin the movie, click on the arrow icon under the Movie header. While the movie is running, it can be paused or stopped at any point for closer examination of the time series laid out thus far. A movie use case of Ebola 2014 outbreak data has been shown in

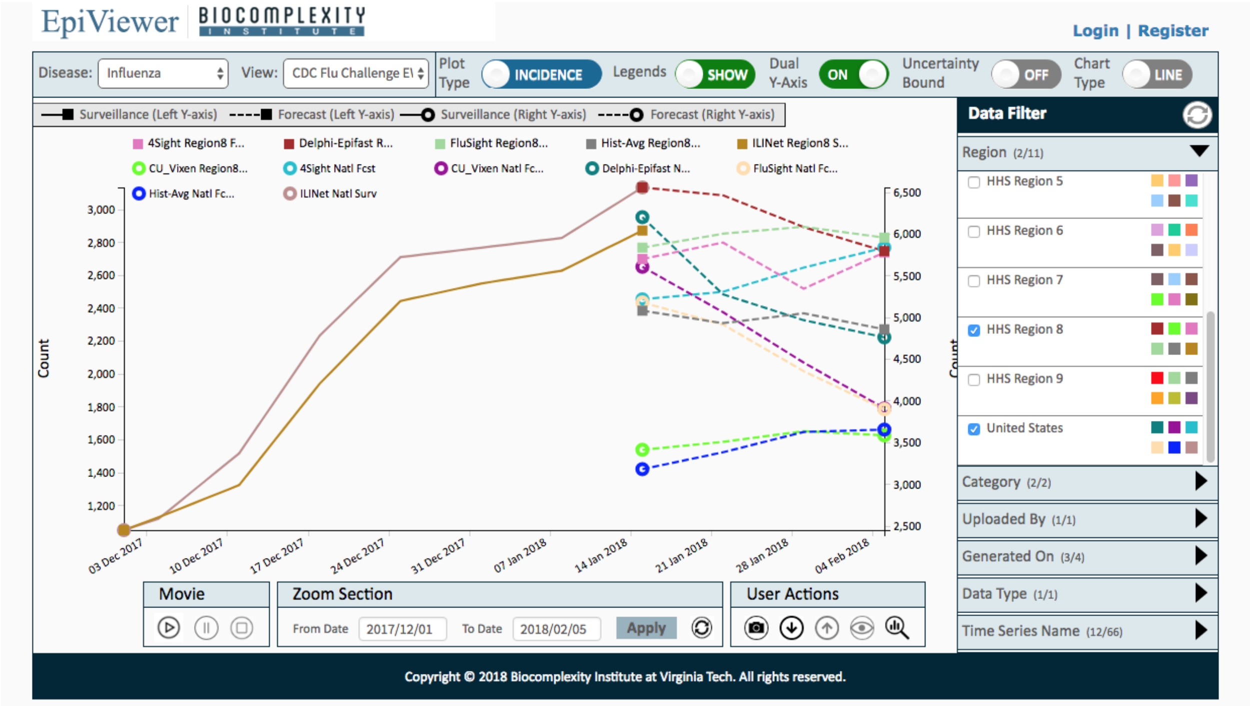

Figure 4: **Distributing time series across dual y-axes.** If the user is having trouble interpreting a time series because it appears flat compared to other time series on the canvas, toggling the Dual Y-axis switch to “On” will add a second, separately scaled y-axis to improve visualization of the time series.

Figure 5 for the Sierra Leone region. The canvas has 3 surveillance curves serving as ground truth. Then, data was collected from various organizations to see how each of them was predicting the outbreak, given the surveillance curve. Each snap shows plots laid out in the order of timestamp. It is interesting to see that some organizations' forecasts could predict the nature of the epidemic.

Download data: Users can download the loaded view's underlying time series and epi-features to CSV format for evaluation outside of the EpiViewer system. The down arrow under the User Actions header will generate a zip file containing all of the plots, each in their own CSV file, as well as a CSV file with a list of the performance metrics.

Snapshot feature: The Snapshot feature under User Actions allows the user to download a picture of the graph. This can then be sent to other users, or used in documentation or publications.

Epi-features: On clicking the 'analyze' button in the 'User Actions' section, a pop-up display will present three epidemiological statistics - peak value and time, first take-off value and time, and total count - for every time series present in the canvas area. This layout depicts a comparative view of these values across the time series. An example of this feature can be viewed in Figure 6.

Supplementary features

EpiViewer has a user management system which tracks users' uploaded graphs and views. Users can upload graphs and perform view manipulations only if they are logged in. The users can provide input about the application or any issues they face by clicking on the Feedback link next to the

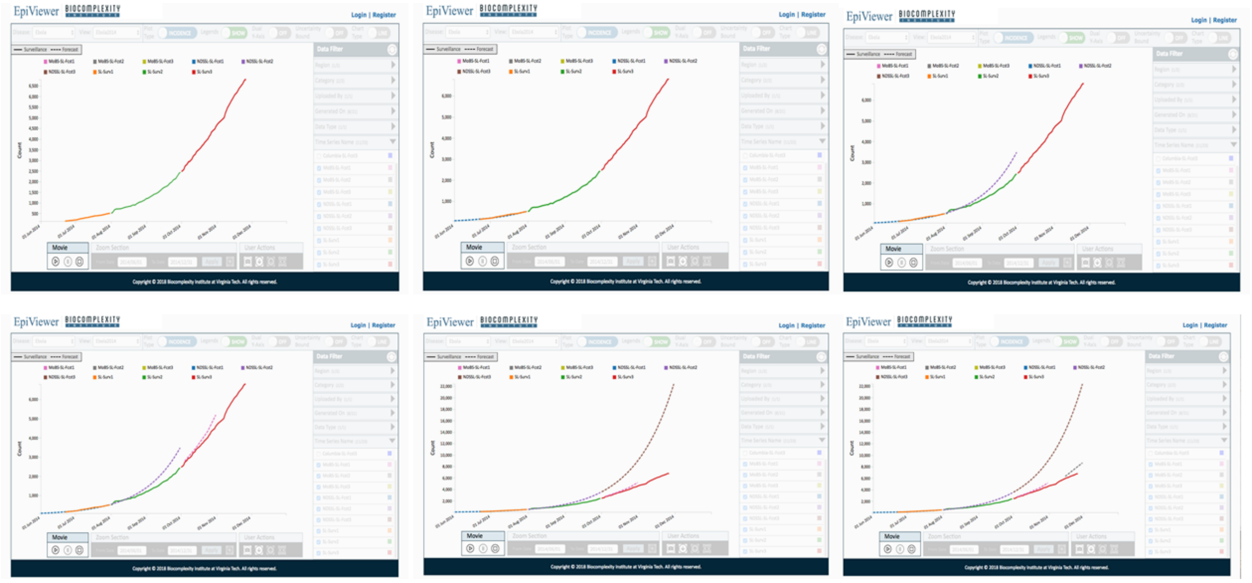

Figure 5: Movie of the 2014 Ebola outbreak progression. This figure shows how data obtained over time shows information about the outbreak.

Logout link on the upper right-hand corner of the display.

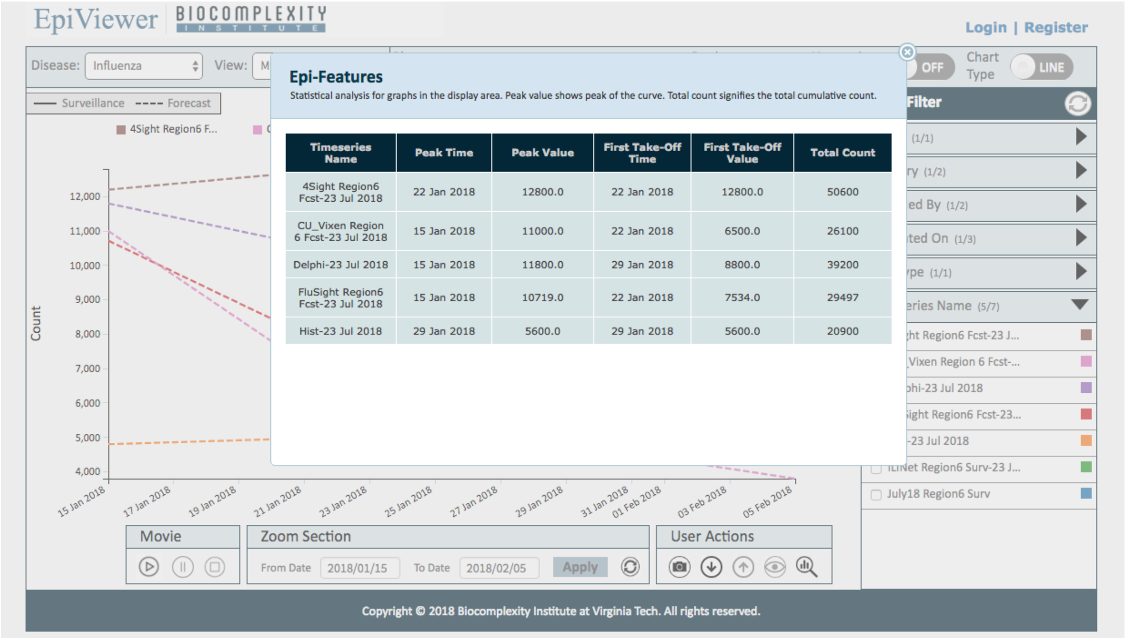

Figure 6: **Epi-Features (Performance Metrics)** Users can view performance metrics of the time series on the canvas to try to assess the quality of the data sets. For example, if the viewer believes the peak value of a time series is too high, or that its first take-off value is too early in the season, they may give more credence to a different forecast with better characteristics.
