# Supplementary material for: EpiViewer: an epidemiological application for exploring time series data
Source: BMC Bioinformatics. 2018 Nov 22;19:449. doi: 10.1186/s12859-018-2439-0 (PMC6251172; doi:10.1186/s12859-018-2439-0)
Supplement: Supplementary file 2 — Exercise for EpiViewer Focus Group. (PDF 341 kb) [file 12859_2018_2439_MOESM2_ESM.pdf]

Exercise for EpiViewer Focus Group

Overview: An instructor will give a quick overview of EpiViewer: what it is, and what problem it was designed to solve. Users will then have the opportunity to try out the system and give us feedback. EpiViewer offers the following features.

- EpiViewer makes it possible for users to view and share EpiCurve time series data plotted on a graph for analytical purposes. The system is preloaded with Influenza data from EpiCaster and Ebola data from various sources during the 2014 crisis in West Africa.
- Users can create their own "Views" to group time series in logical ways; users can also edit or delete their views.
- Users can make their views public in order to share their data with other researchers.
- Users may upload time series data in CSV format into their private views, or include time series from other public or private views.
- They may edit their own time series data or delete it from the system.
- Filtering capabilities allow users to temporarily add and remove time series plots from the view display.
- Users can "zoom in" on a specific date range instead of viewing the entire plot.
- The "Movie" feature allows users to see the time series laid out on the view in sequential order to see how epidemic surveillance and forecast data have evolved over time.
- Users can download the time series from a view in csv format.
- A "Snapshot" feature allows images of the view to be downloaded and shared, or included in papers or other documents.

User exercises:

Please go to http://epics.vbi.vt.edu/EpiViewer/epiviewer.html to attempt the exercises outlined on the following pages. It would be helpful if you could record the start and end time for each exercise.

You may ask questions, but one metric we are trying to track is the intuitiveness of the interface, so please only ask a question if you are truly stuck.

When you complete the exercises, please go to https://epics.vbi.vt.edu/wisdmmturk/epiviewerFocusGroup/ for some final evaluation questions.

1. Create a user account and log in; if you want to remain anonymous, make up a name.

   Time started:________________________ Time completed: ___________________________

2. Load the Ebola 2014 public view.

   Time started:________________________ Time completed: ___________________________

3. Filter the data so only Liberia Forecast data from NDSSL is shown.

   Time started:________________________ Time completed: ___________________________

4. Use the zoom feature to limit the view to December 1, 2014 – December 31, 2014.

   Time started:________________________ Time completed: ___________________________

5. Take a snapshot of the graph and save it to the drive.

   Time started:________________________ Time completed: ___________________________

6. Reset the zoom feature to show the full range of the Ebola View again.

   Time started:________________________ Time completed: ___________________________

7. Use the movie feature to see how NDSSL's forecasts changed over time.

   Time started:________________________ Time completed: ___________________________

8. Create your own Ebola view; include some of the public Ebola time series in the view.

   Time started:________________________ Time completed: ___________________________

9. Upload a time series into your Ebola view (time series data will be provided.)

   Time started:________________________ Time completed: ___________________________

10. Remove one of the public time series from your view.

    Time started:________________________ Time completed: ___________________________

11. Download the data from your view.

    Time started:________________________ Time completed: ___________________________
