# Supplementary material for: EpiViewer: an epidemiological application for exploring time series data
Source: BMC Bioinformatics. 2018 Nov 22;19:449. doi: 10.1186/s12859-018-2439-0 (PMC6251172; doi:10.1186/s12859-018-2439-0)
Supplement: Supplementary file 3 — List of Questions for EpiViewer Focus Group Evaluation. (PDF 193 kb) [file 12859_2018_2439_MOESM3_ESM.pdf]

List of Questions for EpiViewer Focus Group Evaluation

Part 1: Demographic Information

1. Sex:
   (a) Male
   (b) Female
   (c) Other

2. Area of Citizenship: ___________
   (a) North America
   (b) South America
   (c) Europe
   (d) Asia
   (e) Africa
   (f) Australia

3. Which of the following best represents your ethnic group?
   (a) Caucasian
   (b) Asian or Asian American
   (c) Hispanic or Latino/Latina
   (d) Bi- or Multi- Racial
   (e) Black or African American
   (f) Native American or Alaskan Native
   (g) Native Hawaiian or other Pacific Islander
   (h) Other

4. I am (please select one of the following options):

   (a) an undergraduate student

   (b) a masters student

   (c) a doctoral student

   (d) a postdoctoral associate

   (e) a faculty member

   (f) a researcher

   (g) other: ___________________________

5. Please indicate your main area of study and/or research: ___________________________

6. Have you used any tool similar to EpiViewer prior to this evaluation? Yes/No.

   If yes, please specify: ___________________________

Part 2: User Evaluation

Below are a number of statements regarding your experience with EpiViewer. Please read each one and indicate to what extent you agree or disagree with each statement for questions 1-11.

1= Strongly agree 2= Agree 3= Neutral 4= Disagree 5= Strongly disagree

1. EpiViewer is easy to use.
2. EpiViewer requires only a few steps to create views.
3. EpiViewer requires only a few steps to add time series to views.
4. EpiViewer filters are intuitive and easy to navigate.
5. EpiViewer help functionality is informative and easy to understand.
6. EpiViewer provides most of the functionality that users expect it to have.
7. EpiViewer provides clear and helpful error messages, if any.
8. It is easy to understand the inputs requested by EpiViewer.
9. It is easy to specify the inputs requested by EpiViewer.
10. It is easy to understand the results (outputs) generated by EpiViewer.
11. EpiViewer could be a helpful tool in research studies.
12. How likely are you to use a tool like EpiViewer in your future research?

    (a) Very likely
    (b) Somewhat likely
    (c) Not sure
    (d) Somewhat unlikely
    (e) Unlikely

13. What username did you use when applying for an account?
14. Did you encounter any errors while using EpiViewer? What were they?
15. What improvements could be made to EpiViewer to make it easier to use?
16. What new features would you like to see in EpiViewer?
17. Please share any additional comments you have about EpiViewer.
