# Supplementary material for: EpiViewer: an epidemiological application for exploring time series data
Source: BMC Bioinformatics. 2018 Nov 22;19:449. doi: 10.1186/s12859-018-2439-0 (PMC6251172; doi:10.1186/s12859-018-2439-0)
Supplement: Supplementary file 4 — Application Web Services. (PDF 73 kb) [file 12859_2018_2439_MOESM4_ESM.pdf]

Additional File : Application Web Services

This document describes the important web services of the application. The back end of the application (Tier 2) is structured using Representational State Transfer (REST) web services and the Hibernate Java framework. This architectural style considers data and functionality as resources accessed using Uniform Resource Identifiers (URIs), typically links on the web. We use the Jersey RESTful Web Services framework, an open source framework for developing RESTful Web Services in Java. Data formatted in JavaScript Object Notation (JSON) is used for communication between the tiers.

Services

Table 1 shows all the services of the EpiViewer application. Important services have been described following the table.

Table 1: List of services.

ServiceLayer	ServiceName
User Management	addUser()<br>authenticateUser()<br>logoutUser()<br>getAllUsers()<br>saveFeedback()
View (workspace)	getViews()<br>createView()<br>updateView()<br>deleteView()<br>getGraphsByView()<br>downloadGraphData()
Metadata	getAllRegions()<br>getAllDiseases()<br>getAllDataTypes()<br>fetchAllDiseasesCreatedByUser()
Timeseries	getGraphsByView()<br>addGraph()<br>updateGraph()<br>deleteGraphData()<br>uploadGraph()<br>getMetrics()<br>getGraphSelectionForView()<br>getCumulativeDataForBarGraphs()

createView

Create a new view/workspace to load graphs. This feature is available once you have an account with EpiViewer. The service endpoint can be used to create a view external to the application (e.g. through a script).
Parameters

Name	DataType	Description
view	View object	Information wrapped in a object for creation of a new view

Response

Name	DataType	Description
response	String	A JSON object converted to a string containing 3 fields:<br>Id: Unique identifier for the view.<br>DiseaseId: Disease to which the view belongs.<br>Status: Message describing SUCCESS or FAILURE for view creation

updateView

Updates an existing view/workspace details. The graphs in the view will not be altered unless the view is deleted.
Parameters

Name	DataType	Description
view	View object	This object contains the new information to be updated

Response

Name	DataType	Description
response	String	A JSON object converted to a string containing 2 fields:<br>Id: Unique identifier of the view.<br>Status: Message describing SUCCESS or FAILURE for view updating

downloadGraphData

Downloaded all graphs in the given viewId in csv format. The csv files contain all the metadata attributes and the actual data.
Parameters

Name	DataType	Description
viewId	Number	The unique view identifier for which you wish to download data

Response

Name	DataType	Description
data	Response	A class used to build Response instances that contain metadata instead of or in addition to an entity. It is a MIME attachment with the content type 'application/octet-stream' is a binary file.

addGraph

Add a time series and its tagged metadata into the database. This service is called when you upload a graph from the user interface. The updateGraph() service is called if you have to edit the metadata fields of the time series. The time series data itself cannot be altered.
Parameters

Name	DataType	Description
HTML elements data	Multipart form data	Multipart/form-data allows entire files to be included in the data.

Response

Name	DataType	Description
response	String	A JSON object converted to a string containing 4 fields:<br>Id: Unique identifier for the view.<br>DiseaseId: Disease to which the view belongs.<br>Status and Message: String describing SUCCESS or FAILURE for graph upload

uploadGraph

Add a time series and its tagged metadata into the database. This service is called external to the system. The service endpoint can be called by a script in any language. The url and formatted JSON object should be invoked to add the graph into the system. This service is used by BSVE's event trackers and data sources to import data. This API facilitates uploading multiple timeseries (should belong to a single view) in the system. The updateGraph() service can be used from the UI if you have to edit the metadata fields of the time series. The time series data itself cannot be altered.
Parameters

Name	DataType	Description
inputJSON	A JSON object containing fields: data, username, graphName, fileName, description, disease region, plotType, categoryType dataType, dateFormatType forecastedOnDate, graphVisibility	JSON should be converted to string and then passed as input to the service call.

Response

Name	DataType	Description
response	String	A JSON object converted to a string containing 5 fields: viewId, diseaseId, diseaseName, status, message

getMetrics

This service calculates epi features for every graph selected in the filters or present in the canvas area. All the epi features are calculated on the fly every time the user clicks the epi features button in the 'user actions' panel.

Parameters

Name	DataType	Description
graphIds	String	Comma separated list of graph ids

Response

Name	DataType	Description
metricJSON	JSON Array	A JSON array where each JSON object contains: graphId, graphName, peakValue, peakTime, totalCount, firstTakeOffValue, firstTakeOffTime

getGraphSelectionForView

Get all the graphs / time series required while creating new view or editing an existing view. This is an important service since users have access to their own private collection of workspaces and then publicly available workspaces. A logged in user is entitled to choose graphs from any of these collections into a new view or add them into an existing view.

Parameters

Name	DataType	Description
diseaseId,viewId,username	Number,Number,String	Attributes for graph selection in a view

Response

Name	DataType	Description
graphs	Map[String, List[GraphMetadata]]	A dictionary containing graphs from the respectives user's public and private views. If editing a view, then the existing graphs in the view are also shown. Three keys are present in the map, namely, 'publicGraphs', 'privateGraphs', 'existingGraphs'
